# Seminal Papers in Urology: Darolutamide and survival in metastatic, hormone-sensitive prostate cancer

**DOI:** 10.1186/s12894-024-01507-7

**Published:** 2024-07-01

**Authors:** Claris Oh, Michael O’Callaghan

**Affiliations:** 1 Urology Unit, Flinders Medical Centre, Adelaide, Australia; 2 Discipline of Medicine, Adelaide University, Adelaide, Australia; 3 College of Medicine and Public Health, Freemasons Centre for Male Health and Wellbeing, Flinders University, Adelaide, Australia

**Keywords:** Prostate cancer, Daralutamide, Metastatic, Survival

## Abstract

The ARASENS trial recruited 1306 men with metastatic hormone sensitive prostate cancer. It investigated the effect of androgen deprivation therapy (ADT) and systemic therapy docetaxel in combination with a third novel drug – daralutamide, compared with placebo on overall survival. Triple therapy with ADT, docetaxel and darolutamide resulted in improved overall survival rates as compared with ADT, docetaxel and placebo (HR 0.68; 95% CI, 0.57–0.80; *p* < 0.001). The side effect profile for both treatments was similar. This randomised, double blinded, placebo controlled study, was assessed to have a low risk of bias using the Cochrane Risk of Bias 2 tool.

## The clinical problem

Prostate cancer is a common diagnosis, and its prevalence is expected to increase as the population ages. The disease is also the sixth leading cause of death amongst men [1]. This is despite having significant advances in treatment of prostate cancer in the recent years, with development of new therapies and drugs approved for treatment of metastatic prostate cancer. Current guidelines by European Association of Urology (EAU) [2] and American Urological Association (AUA) [3] recommend offering all patients with metastatic hormone sensitive prostate cancer (mHSPC) a combination of androgen-deprivation therapy (ADT) plus systemic therapy docetaxel. There have also been previous trials supporting the addition of an androgen-receptor pathway inhibitor like abiraterone [4], enzalutamide [5] or apalutamide [6] to ADT. Triple therapy with ADT, docetaxel and the above-mentioned androgen-receptor pathway inhibitors had also been investigated, with varying results [7, 8].

## The study

Darolutamide is a novel drug which has been shown to have significant benefits to survival rates in non-metastatic, castrate-resistant prostate cancer. Combinations with ADT were improved survival rates in comparison to ADT alone [9]. Through a randomised, double-blind, placebo-controlled trial, Smith et al. compared the survival rates of standard therapy of ADT plus docetaxel, with the addition of androgen-receptor pathway inhibitor darolutamide to the two drugs. The results were published in The New England Journal of Medicine in March 2022 [10].

Adult patients above the age of 18, with confirmed prostate cancer through histology or cytology and radiologically proven metastasis were eligible to enter the trial. Other inclusion criteria included having an Eastern Cooperative Oncology Group status of 0 to 1.

The primary outcome measure of overall survival was defined as the duration from time of randomisation to time of death, regardless of cause. Secondary outcome measures included time to progression of disease to being castrate resistant or initiation of systemic chemotherapy, as well as less subjective outcomes such as worsening symptoms measured through pain scores.

All patients recruited in the study underwent ADT or orchidectomy within 12 weeks before randomisation, and also had received six cycles of docetaxel. Patients also received doses of oral steroids to prevent hypersensitivity reactions and fluid retention according to clinician discretion.

Randomisation to receiving either darolutamide or placebo was in a 1:1 ratio, and was adjusted for metastasis stage according to the TNM system and serum levels of ALP. This was conducted by separate personnel, through a computer-generated randomisation list. The study aimed for 90% power to detect a 25% decrease in risk of death in the darolutamide group versus placebo group, and achieved this by recruiting total of 1306 patients across multiple sites in multiple countries. Patients were assessed based on the treatment they received.

There was equal distribution of the baseline characteristics between the two study groups which suggests that randomisation was implemented well. Only one and three patients were excluded from full analysis and safety analysis set respectively.

## Summary of findings

There was a 32.5% reduction in risk of death for patients who received darolutamide as compared to those who received placebo, representing a significant improvement in overall survival (hazard ratio, 0.68; 95% confidence interval [CI], 0.57 to 0.80; *P* < 0.001). Overall survival rate at four years was 62.7% in the darolutamide group and 50.4% in placebo group. The patients who were allocated to receive darolutamide with ADT and docetaxel also performed better in secondary outcomes than those in the placebo group.

Adverse event rate was similar in the two groups, with the most commonly reported being alopecia, followed by neutropenia, fatigue and anaemiaIn the darolutamide group 13.5% of patients with adverse event led to their discontinuation of the drug and only 10.5% in placebo group.

## Assessment of evidence

The ARASENS phase 3 trial discussed in this paper is a large, multicentre, international study involving 23 countries and spanning five continents. There is good representation of not only race and cultures but also a wide range of healthcare systems.

There was a low risk of bias with appropriate steps taken to ensure adequate blinding of the patients, investigators and sponsors. Patients were analysed under their Intention to Treat groups and there were minimal missing data to affect the study results significantly. According to the Cochrane risk-of-bias assessment [11], the study has a low risk of bias in all domains. Although it should be acknowledged that the study was sponsored by Bayer and Orion Pharm, which might represent a potential risk for bias. The sponsors were involved in data analysis, interpretation, authorship and manuscript writing.

## Future research

Future efforts could be directed to comparing the overall survival rates between having double therapy darolutamide and ADT versus ADT and docetaxel. Given that most of the adverse effects reported in this study are well known side effects from docetaxel, patients could potentially benefit from a reduction in the number of chemotherapy drugs they receive.

The trial could also be expanded to include patients of poor ECOG scores. This additional data is likely to become available in real world evidence studies (noting the non-randomised nature of this study type) and will be useful in assessing the generalisability of the study results. In addition, comparing adverse effects with alternative second generation androgen receptor inhibitors will be important, particularly in the context of similar efficacy [12].

ADT treatment of prostate cancer patients is becoming increasingly complex. This study demonstrates that darolutamide is beneficial in the setting of metastatic disease, and adds to data showing a benefit to men with non-metastatic castrate resistant disease [13]. These two patient settings are common targets for second generation androgen deprivation medications. To date, ADT monotherapy treatment of low risk and intermediate disease has not been demonstrated beneficial, and has limited application in high risk settings. Increasing focus on accurate diagnostic staging, stratification and medication combinations particularly minimising adverse events will likely characterise this field in the near term.

## Data Availability

This paper uses no primary data.

## References

[1] Culp MB, Soerjomataram I, Efstathiou JA, Bray F, Jemal A (2020). Recent global patterns in prostate Cancer incidence and mortality rates. Eur Urol.

[2] EAU Guidelines. Edn presented at the EAU Annual Congress Amsterdam. 2022. ISBN 978-94-92671-16-5.

[3] Lowrance WT, Breau RH, Chou R (2021). Advanced prostate Cancer: AUA/ASTRO/SUO Guideline PART I. J Urol.

[4] Tan PS, Aguiar P, Haaland B, Lopes G (2018). Addition of abiraterone, docetaxel, bisphosphonate, celecoxib or combinations to androgen-deprivation therapy (ADT) for metastatic hormone-sensitive prostate cancer (mHSPC): a network meta-analysis. Prostate Cancer Prostatic Dis.

[5] Armstrong AJ, Szmulewitz RZ, Petrylak DP (2019). ARCHES: a Randomized, Phase III Study of Androgen Deprivation Therapy with Enzalutamide or Placebo in Men with metastatic hormone-sensitive prostate Cancer. J Clin Oncology: Official J Am Soc Clin Oncol.

[6] Chi KN, Chowdhury S, Radziszewski P, et al. TITAN: a randomized, double-blind, placebo-controlled, phase 3 trial of apalutamide (ARN-509) plus androgen deprivation therapy (ADT) in metastatic hormone-sensitive prostate cancer (mHSPC). Ann Oncol. 2016;27(suppl6). 10.1093/annonc/mdw372.54.

[7] Davis ID, Martin AJ, Stockler MR (2019). Enzalutamide with Standard First-Line therapy in metastatic prostate Cancer. N Engl J Med Jul.

[8] Fizazi K, Carles Galceran J, Foulon S (2021). LBA5 A phase III trial with a 2 × 2 factorial design in men with de novo metastatic castration-sensitive prostate cancer: overall survival with abiraterone acetate plus prednisone in PEACE-1. Ann Oncol.

[9] Fizazi K, Shore N, Tammela TL (2020). Nonmetastatic, castration-resistant prostate Cancer and survival with Darolutamide. N Engl J Med.

[10] Smith MR, Hussain M, Saad F (2022). Darolutamide and Survival in Metastatic, hormone-sensitive prostate Cancer. New Engl J Med 2022/03/24.

[11] Sterne JAC, Savović J, Page MJ, Elbers RG, Blencowe NS, Boutron I, Cates CJ, Cheng H-Y, Corbett MS, Eldridge SM, Hernán MA, Hopewell S, Hróbjartsson A, Junqueira DR, Jüni P, Kirkham JJ, Lasserson T, Li T, McAleenan A, Reeves BC, Shepperd S, Shrier I, Stewart LA, Tilling K, White IR, Whiting PF, Higgins JPT (2019). RoB 2: a revised tool for assessing risk of bias in randomised trials. BMJ.

[12] Chen X, Wang Q, Pan Y (2023). Comparative efficacy of second-generation androgen receptor inhibitors for treating prostate cancer: a systematic review and network meta-analysis. Front Endocrinol (Lausanne).

[13] Fizazi K, Shore N, Tammela TL (2019). Darolutamide in Nonmetastatic, Castration-resistant prostate Cancer. N Engl J Med.

